# Focus on Function in Hospitalized Persons With Dementia: The Impact of Hospital-Acquired Complications

**DOI:** 10.1093/geroni/igaa057.2750

**Published:** 2020-12-16

**Authors:** Marie Boltz, Lorraine Mion

## Abstract

Persons with dementia (PWD) are two-three times more likely to be hospitalized as persons without dementia and comprise one fourth of hospitalized older adults. Hospitalization often has a dramatic impact upon the health and disposition of the older PWD. They are at increased risk for hospital acquired complications (HAC) such as functional decline, behavioral symptoms of distress, and delirium, all of which contribute to increased disability, mortality, and long-term nursing home stays. Despite the unprecedented number of PWD admitted to acute care, little attention has focused on their specialized needs and HAC, and how they impact functional recovery. The purpose of this symposium is to describe the incidence of common HACs, and factors that influence their occurrence and presentation in PWD. Utilizing baseline findings from the Family-centered Function-focused Care (Fam-FFC) trial, the presentations will address this objective and discuss the ramifications for functional and cognitive post-acute recovery in PWD. The first presentation will describe the incidence and pharmacologic management of pain in PWD, and its association with common HACs. The second presentation will describe physical activity in PWD on medical units and the validity of the Motionwatch8 actigraphy. The third session will describe differences in common HACs between white and black PWD. The final presentation will examine function-focused goals developed in collaboration with family caregivers and patients, and the functional outcomes associated with goal attainment. Our discussant, Dr. Lorraine Mion, will synthesize the research findings and lead a discussion of future directions for policy and practice in dementia-capable acute care.

